# Current challenges in metastasis research and future innovation for clinical translation

**DOI:** 10.1007/s10585-021-10144-5

**Published:** 2022-01-24

**Authors:** Amelia L. Parker, Madeleine Benguigui, Jaime Fornetti, Erica Goddard, Serena Lucotti, Jacob Insua-Rodríguez, Adrian P. Wiegmans, Early Career Leadership Council of the Metastasis Research Society

**Affiliations:** 1 Matrix and Metastasis Lab, Kinghorn Cancer Centre, Garvin Institute of Medical Research, Darlinghurst, NSW 2010 Australia; 2 St Vincent’s Clinical School, UNSW Sydney, Sydney, 2052 Australia; 3 Cell Biology and Cancer Science, Rappaport Faculty of Medicine, Technion-Israel Institute of Technology, 31096 Haifa, Israel; 4 Department of Oncological Sciences, Huntsman Cancer Institute, University of Utah, Salt Lake, UT USA; 5 Public Health Sciences Division/Translational Research Program, Fred Hutchinson Cancer Research Center, Seattle, WA USA; 6 Children’s Cancer and Blood Foundation Laboratories, Departments of Pediatrics, and Cell and Developmental Biology, Drukier Institute for Children’s Health, Meyer Cancer Center, Weill Cornell Medicine, NY New York, USA; 7 Department of Physiology and Biophysics, Department of Biological Chemistry, Chao Family Comprehensive Cancer Centre, University of California, Irvine, CA USA; 8 Cancer and Ageing Research Program, Centre for Genomics and Personalised Health, Queensland University of Technology (QUT), Translational Research Institute, Woolloongabba, QLD 4121 Australia

**Keywords:** Metastasis, Liquid biopsy, Latency, Dormancy, Microenvironment, Immunotherapy

## Abstract

While immense strides have been made in understanding tumor biology and in developing effective treatments that have substantially improved the prognosis of cancer patients, metastasis remains the major cause of cancer-related death. Improvements in the detection and treatment of primary tumors are contributing to a growing, detailed understanding of the dynamics of metastatic progression. Yet challenges remain in detecting metastatic dissemination prior to the establishment of overt metastases and in predicting which patients are at the highest risk of developing metastatic disease. Further improvements in understanding the mechanisms governing metastasis have great potential to inform the adaptation of existing therapies and the development of novel approaches to more effectively control metastatic disease. This article presents a forward-looking perspective on the challenges that remain in the treatment of metastasis, and the exciting emerging approaches that promise to transform the treatment of metastasis in cancer patients.

## Introduction

Advances in understanding the key features of primary tumors have relied on innovation across specialized fields, culminating in improved clinical staging and patient survival rates. These advances have also enhanced our understanding of secondary tumor formation, or metastasis, that remains the major cause of cancer-related deaths. Metastasis is defined as the process in which cancer spreads from the primary tumor and establishes at anatomically distinct sites. While tremendous technology-driven advances in our understanding of the metastatic process are revealing promising targetable mechanisms, improving the outcomes of patients with metastatic disease remains a significant challenge. In this perspectives article we identify opportunities for emerging fields of investigation that have the potential to fundamentally revolutionize not only our understanding of the metastatic process, but also the way in which metastasis is treated.

Despite advances in cancer detection and treatment, residual disseminated disease remains present but undetected in a considerable proportion of patients whose primary tumor has been successfully treated. This residual disease can be present as micrometastases, defined as multicellular secondary tumor cell clusters, or as disseminated single tumor cells (DTCs) that are currently too small to detect in clinical diagnostic scans and persist as potential sources of subsequent metastatic relapse [1]. The latency period between initial diagnosis and metastatic recurrence varies between months and years and a number of models have been proposed to explain these dynamics. One predominant model is that disseminated cells undergo a period of cellular dormancy prior to awakening and giving rise to overt metastases [2, 3]. Alternative models propose that the growth rate of disseminated cells remains relatively constant and instead it is the balance between proliferation and cell death in the disseminated cells that constrains the emergence of overt metastases until proliferation rates dominate [4]. The maturation models suggests that disseminated tumour cells must first acquire further genomic alterations that enable their overt growth at secondary sites, and this maturation process results in delayed formation of detectable secondary tumors following dissemination [4]. It is likely that latency periods and the evolution of metastatic disease results from mixtures of these models, and that the contribution of each model to patient outcome differs between tumour types, secondary sites and is influenced by multiple host factors. Highly variable latency periods present a challenge in monitoring patients for metastatic emergence. Furthermore, current diagnostic approaches lack the sensitivity to detect this minimal residual disease, and as a result, the temporal dynamics of tumor cell dissemination and the overall burden of disseminated disease remains unclear for most cancer types. Yet, while the presence of substantial micrometastatic disease is suggestive of a high risk of relapse, not all patients will develop overt metastases from these disseminated cells. Monitoring and modelling tumor latency dynamics, in particular dormancy and reawakening, using patient avatars is crucial for understanding relapse mechanisms and predicting patient populations at risk.

The processes that govern the dynamics of tumor cell dissemination from the primary site, seeding at secondary sites, and ultimately their outgrowth into overt metastases are emerging as complex, dynamic and spatially compartmentalized interactions between cancer cells and the local tissue microenvironment [5–7]. The additional impacts of host and environmental factors adds further complexity to the myriad regulators of tumor progression, confounding our ability to accurately predict the trajectory of each patient’s cancer and the most effective treatment. This intra- and inter-individual complexity heralds an era of precision medicine that exploits our understanding of these factors to tailor therapies that specifically target metastatic disease.

**Fig. 1:**
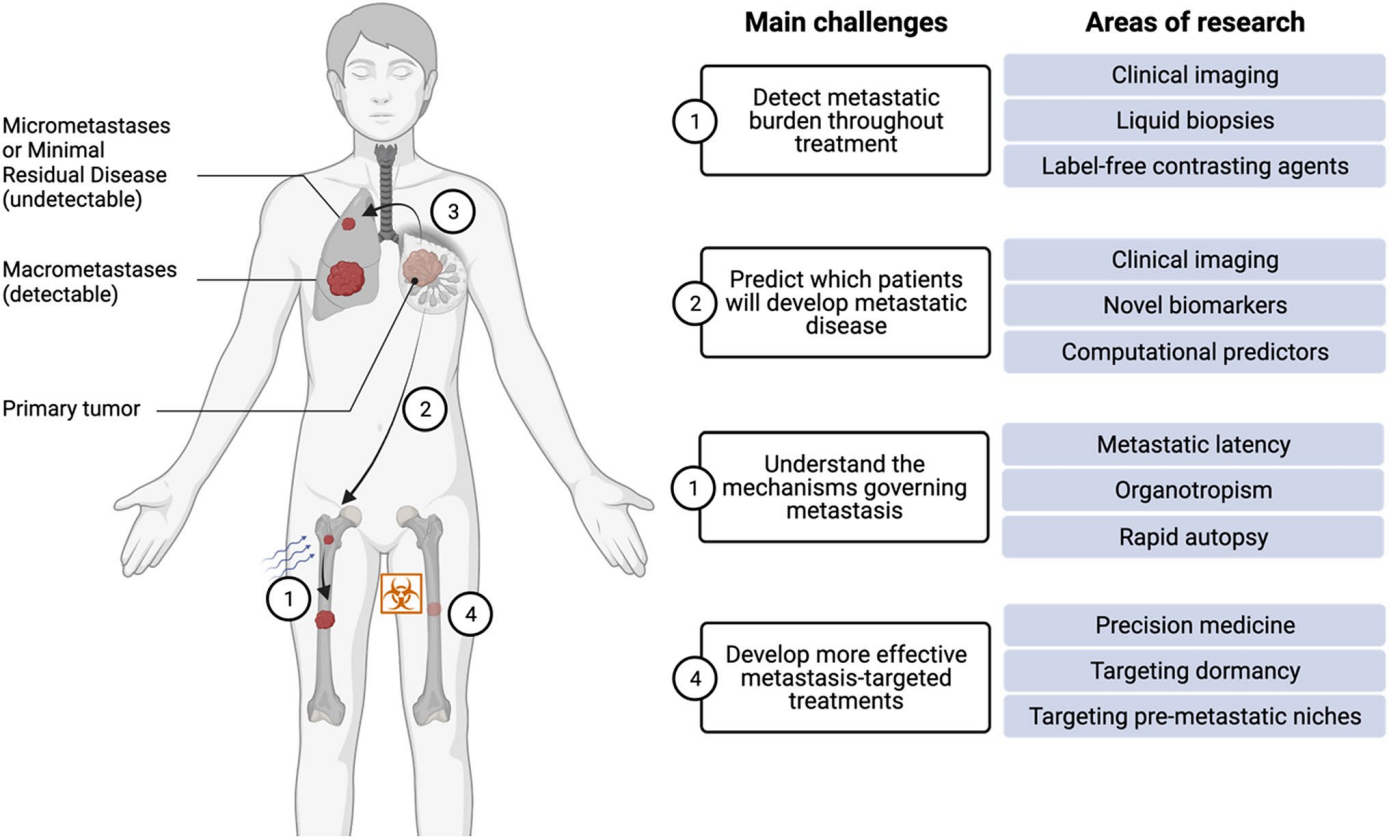
Current challenges in the management of metastatic disease and areas of research addressing these challenges. Created with BioRender.com

To achieve a precision medicine framework that improves the outcome of patients with metastatic disease, we must understand the collective influence of cancer cell intrinsic, tumor microenvironmental, host, and environmental factors on tumor behavior before translating this knowledge into targeted therapies. Growing evidence indicates that despite the unambivalent utility of chemotherapy in successfully treating the primary tumor and improving patient outcomes, it has been demonstrated that some chemotherapies and dosing regimes can accelerate metastatic progression in some in vivo models in a context-specific manner [8, 9] and further study is required to determine if similar effects are seen in patients. Understanding how primary tumor treatments affect metastatic dissemination provides an opportunity to more effectively implement existing treatments to also inhibit metastasis, while also suggesting that novel therapeutic approaches may be required to specifically target metastasis. Precision medicine approaches that take into account the dynamics of metastatic latency, outgrowth and response to primary tumor therapies for those metastases that progress will revolutionize the clinical management and outcomes for cancer patients. As depicted in Fig. 1, major challenges to realizing improved outcomes in metastatic disease can be summarized as:

1. Detecting and quantifying the metastatic burden throughout treatment.
2. Predicting which patients will develop overt metastatic disease.
3. Understanding the mechanisms governing tumor metastasis.
4. Developing more effective metastasis-targeted treatments.

**Fig. 2:**
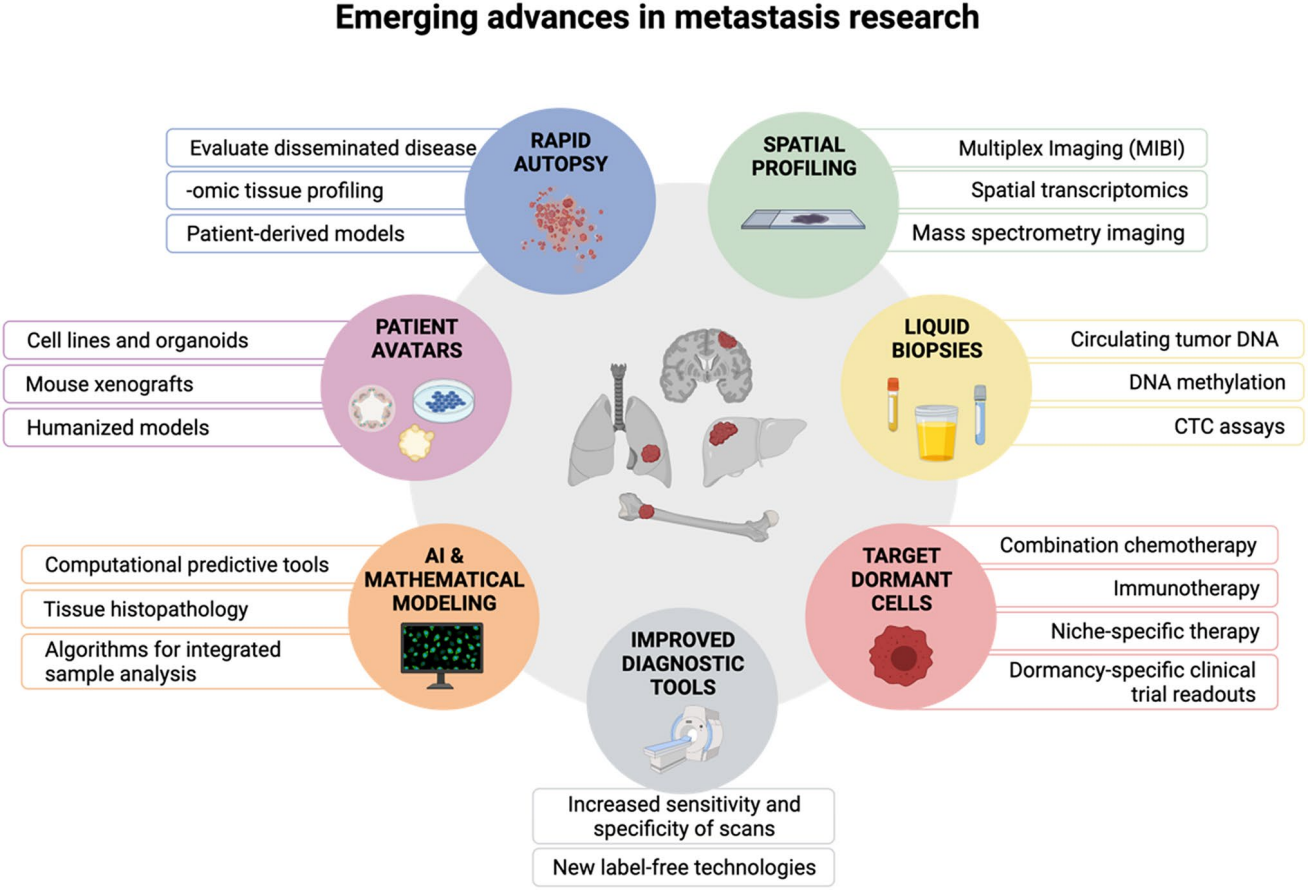
Emerging advances in metastasis research. Created with BioRender.com

Emerging advances overcoming these challenges have the potential to transform the clinical management of metastatic disease across cancer types (Fig. 2).

## Dynamically defining metastatic burden during treatment

### Current approaches: improving pathological assessments

In order to improve the management of metastatic disease, it is imperative to obtain an accurate picture of when metastatic dissemination occurs, and how this process defines the risk profile for individuals both at diagnosis and throughout treatment. While great gains have been made in mapping the dynamics of tumor progression in many cancer types, much work remains to create a comprehensive temporal map of metastatic progression for each tumor type. To date, advancements in accurately assessing disseminated tumor burden in patients have been hampered by the limited sensitivity of radiological diagnostic scans for micrometastatic disease. Lymph node assessment, including sentinel node mapping with pathological assessment and ultrastaging (e.g. cytokeratin staining) as a surrogate for metastatic dissemination, has instead been used as a mainstay of metastatic disease staging in surgically resected cancers [10]. However, the recent integration of deep learning image analysis algorithms, which use multiple tissue features to define the presence of cancer cells, into clinically-established pipelines are improving the sensitivity and reproducibility of pathological metastasis staging [11, 12]. In particular, Convolutional Neural Networks (CNN) have been the most widely studied artificial intelligence (AI) architecture for segmenting pathological images to classify tumour-associated regions of interest [13, 14]. While the large amount of data required to train CNN architectures remains a significant hurdle in developing robust algorithms, these network architectures have been used to develop the most advanced AI algorithms to improve pathological staging, such as those developed from the CAncer MEtastases in LYmph Nodes Challenge (CAMELYON) [15]. The best performing algorithms to come out of this challenge were able to diagnose positive lymph node metastases with improved accuracy and efficiently compared with a panel of 11 pathologists [16]. This has led to the next phase of challenges that test AI capability to identify metastatic tissue in histopathologic scans demonstrating a pooled sensitivity of 82%, specificity of 84% and AUC of 0.90 for identification of tumor metastasis based on a summary of 2620 studies [16]. As the morphological and biochemical characteristics of tissue sites primed for metastatic colonisation become more clearly defined, we propose that the capacity of AI to detect overt metastasis will soon transfer to the ability to detect these pre-metastatic niches with equivalent accuracy. Such improvements in current clinical diagnostic pipelines will enable the early detection of metastatic dissemination to accurately prioritize patients for the most effective treatment.

Despite these improvements, metastatic lymph node assessment, by its nature, only captures cells that are within the lymphatic system and fails to detect cancer cells that have already disseminated to distant organs. Therefore, there is a clear need to improve the sensitivity and specificity of current diagnostic scans and develop label-free technologies that together can provide a whole-body picture of micrometastatic burden to define prognosis. In this regard, cross-platform imaging technologies that extend existing radiographic and radiological imaging technology to detect micrometastatic sites are showing promise [17].

The next phase in developing AI and imaging technology is in the detection of sites primed for metastatic colonization, thereby enabling the earliest assessment of metastatic risk and the opportunity to prevent the establishment of micrometastatic disease. These sites, known as pre-metastatic niches, are regions within distant organs that, under the influence of primary tumor-derived systemic factors, are primed to support the establishment and persistence of metastatic disease [18]. These pre-metastatic niches have now been characterized in the lungs [19–21], liver [22], lymph nodes [23], bone marrow [24] and brain [19] in both pre-clinical models and cancer patients. While the specific features of these niches appear to be tumor- and organ-dependent, vascular leakiness, increased inflammation (e.g. TLR4 activation), alteration of the extracellular matrix (ECM), recruitment of immunosuppressive cells (including macrophages, bone marrow derived cells (BMDCs) and regulatory T cells) as well as the activation and metabolic reprogramming of resident stromal cells are all common features of the pre-metastatic niche [26]. Together, these changes shape the pre-metastatic niche to be more receptive of tumor cell settlement by enhancing nutrient availability, vessel permeability, inflammation and cancer cell migration, survival and adhesion to ECM components at these distant sites. Altered textural features in radiological scans of axillary lymph nodes and metastatic sites that reflect increased matrix deposition or tissue density for example [17, 22], are showing promise in the detection of these pre-metastatic niches [25], as are emerging imaging agents targeted against specific features of the pre-metastatic niche, such as overexpression of the α4β1 integrin receptor [27] or the presence of specific fibronectin isoforms in the ECM [17]. For example, radiomic analysis of the liver parenchyma on presurgical CT scans has been shown to predict the future development of hepatic metastases in colon cancer patients following primary tumor resection [25].

Further progress developing these emerging technologies will facilitate the dynamic monitoring of metastatic dissemination, allowing for rapid adaptation of therapies to maximize clinical responses (Table 1).

### Monitoring metastatic spread during treatment response

Liquid biopsy biomarker detection is a promising complementary approach to image-based detection of metastatic disease. Liquid biopsies are derived from plasma, serum or urine, and their minimally invasive nature makes them amenable to longitudinal tracking of metastatic progression in response to therapy. Detecting circulating tumor cells (CTCs) or their DNA (circulating tumor DNA, ctDNA) in plasma and serum are currently regarded as the most direct methods for assessing disseminated tumor burden using liquid biopsies. The number of CTCs or amount of ctDNA detected after therapy are robust readouts for treatment efficacy and with their superior sensitivity, enable the detection of relapse many months earlier than current radiological imaging procedures allow [28].

Despite the promise of monitoring CTCs as a direct measure of tumor cell dissemination [29], clinical implementation of CTC biomarkers as decision-making tools has been hampered by the scarcity of CTCs in blood specimens, a lack of standardized cell isolation approaches and inadequate sensitivity. CELLSEARCH^TM^, currently the only FDA-approved molecular pathology assay to detect CTCs, has overcome sensitivity limitations by implementing an EpCAM-positivity CTC enrichment step followed by an imaging-based tumor cell detection using cytokeratin and nuclear staining [30, 31]. Although EpCAM+ enrichment and cytokeratin staining is a standard approach in a research setting, growing evidence indicates that EpCAM enrichment likely captures a subset of epithelial tumor cells and may not capture those tumor cells that have undergone a mesenchymal transition during metastatic dissemination [31], thereby limiting the assay’s applicability to specific epithelial cancers. Such approaches to standardize the isolation and enrichment of CTCs will provide insight into the dynamics of tumor cell dissemination as well as the opportunity to identify pro-metastatic features of cancer cells that can be therapeutically targeted [32, 33].

Comparatively, ctDNA biomarker development has leveraged the sequencing revolution to demonstrate superior sensitivity and specificity compared with CTC analysis [29]. CtDNA markers that show considerable promise for clinical translation are mapped to mutated DNA regions corresponding to prevalent cancer drivers and are therefore cancer-type specific. These include mutant adenomatous polyposis coli (*APC*), epidermal growth factor receptor (*EGFR*) and Kirsten rat sarcoma virus (*KRAS*) DNA in the plasma of colorectal cancer patients as indicators of response to conventional and EGFR-targeted therapies, respectively, which are being developed for clinical use [34, 35]. More recently, digital drop PCR pre-amplification and fluorescent probes have been developed into a promising standardized assay that detects ctDNA derived from mutant histone H3-genes in pediatric diffuse midline glioma patients, a tumor type that is generally not surgically accessible [36]. While these highly specific single-gene approaches are valuable for monitoring targeted therapy response, a panel of multiple ctDNA gene targets will be required to capture the burden of DTCs derived from diverse tumor types and highly clonal, genetically heterogeneous tumors. Multi-gene profiling of ctDNA in plasma samples, such as that implemented by MSK-ACCESS (MSK-Analysis of Circulating Cell-free DNA to Evaluate Somatic Status), overcomes the cancer- and subclone-specific nature of these targeted approaches to provide a potential multi-cancer or clonal diagnostic to guide clinical decision making [37]. The application of existing technology to different cell types in liquid biopsies also has the potential to improve ctDNA biomarker performance in detecting disseminated cancer cells derived from heterogeneous tumors. For example, comparative deep sequencing of ctDNA and matched healthy hematopoietic cell DNA from patients with highly clonal lung tumors was more accurate in predicting patient prognosis compared to ctDNA analysis alone [38]. In addition, combining ctDNA biomarkers with existing imaging modalities, such as specialized positron emission tomography - computed tomography (PET–CT), can improve the sensitivity and specificity of micrometastasis detection [38], suggesting that a multifaceted approach may achieve clinical benefit in the near term.

While ctDNA analysis is a promising technology for indirectly detecting DTCs, it cannot indicate their discrete anatomical location, thereby limiting its application as a stand-alone diagnostic tool. Fragmentomics and epigenetic profiling of ctDNA are emerging fields of investigation that have the potential to overcome these limitations by enabling identification of the ctDNA organ of origin, and therefore the primary tumor location [39, 40]. Fragmentomics analysis is founded on the principles that DNA fragmentation occurs in a tissue-specific manner due to the influence of nucleosomal organization, chromatin structure, gene expression, and nuclease content of the tissue of origin, resulting in characteristic organ-specific signatures of ctDNA fragment size, nucleotide motifs at the fragment ends, and the genomic locations of the fragmentation endpoints [40]. Similarly, the detection of aberrant epigenetic methylation patterns, which are more prevalent and penetrant than genetic mutations in ctDNA, provides a more sensitive detection of ctDNA than mutational analysis alone, and also indicates tissue of origin [39]. However, methylome analysis is not yet capable of indicating the location of metastatic sites. With over 20 currently active clinical trials evaluating ctDNA methylation in the diagnosis and monitoring of various cancers (as of April 2021), the development of these ctDNA detection approaches holds immense promise not only in monitoring metastatic dissemination but also in cancer diagnosis.

The monitoring of circulating protein markers and extracellular vesicles derived from cancer cells are also being developed as markers of tumor burden and as surrogates for metastatic disease in tumors that have been surgically resected, for example, CA19-9 in pancreatic cancer [41]. However, circulating levels of primary tumor markers alone are not always predictive of metastatic prognosis, such as biochemical recurrence in prostate cancer as indicated by PSA levels [42]. By specifically detecting secreted factors and extracellular vesicles derived from micrometastatic sites or those that play a critical role in priming distant pre-metastatic niches [20, 22, 24, 43–45] it may be possible to monitor for metastatic propensity and likely secondary sites at earlier stages of recurence. For example, integrin signatures of tumor-derived exosomes orchestrate tumor cell organotropism [46] and, together with other exosomal protein and miRNA cargos, educate resident cells such as Kupffer cells, BMDCs, fibroblasts and endothelial cells to promote metastasis [19, 22, 44–46]. Importantly, high levels of exosomal proteins and miRNAs involved in pre-malignant niche establishment correlate with poor prognosis and higher risk of metastatic disease [19, 22, 44–46]. Therefore, the use of metastatis-specific protein- and RNA-based biomarkers may leverage existing diagnostic technology to enable monitoring of metastatic burden throughout treatment.

**Table 1:** Observational clinical trials to improve metastasis detection and understand risk associations

Modality	Cancer type	Details	Trial ID
Serum and tumor VEGF levels (ELISA)	Colorectal	Testing the association of VEGF levels with presence of lymph node metastases	NCT04145505
Presence of lymph node metastases	Endometrial cancer, cervical cancer	Testing the association between survival and presence of lymph node metastases	NCT04403867
Detection of DTCs by real time PCR	Gastric cancer, pancreatic cancer	Testing real-time PCR assay to detect the presence of DTCs within the peritoneum at the time of surgical resection.	NCT00582062
Detection of DTCs by real time PCR	Prostate cancer	Detection of micrometastases in lymph nodes by real time PCR assay	NCT01615965
Improved optical imaging of metastases in sentinel lymph nodes	Melanoma	Sentinel lymph node imaging using near infrared fluorescence contrast imaging and indocyanine green staining	NCT02142244
CTCs and DNA	Lung cancer, esophageal cancer, gastric cancer, pancreatic cancer, hepatocellular carcinoma, colorectal cancer	Assessing the detection of cancer cells and cancer cell DNA in blood, urine and bone marrow	NCT02838836

### Translating disseminated tumor cell burden into clinical risk measures

While the DTC burden reflects the potential for metastatic disease to develop, the establishment of overt metastasis from CTCs and micrometastases is a highly inefficient process [47]. For example, in breast cancer, less than half of the patients with detectable micrometastases in the bone marrow develop distant recurrence within 10 years [48]. Therefore, the volume of disseminated disease when present as small cell clusters or single cells does not always directly correlate with the incidence of overt metastasis. Seminal studies have begun to dissect the key intrinsic tumor cell features that confer the capacity to form overt metastases, and have shown that these features are present in a specific subset of tumor cells [49]. This highlights a need to understand the cell intrinsic and extrinsic factors that act in concert with the disseminated disease burden to define a patient’s metastatic propensity.

Different secondary sites have different propensities for the development of overt recurrence [50]. For example, lymph nodes, liver, lung, and bone are common metastatic sites across a multitude of primary cancer types, yet overt metastases in skeletal muscle are relatively rare [51]. While the mechanisms underlying these patterns are not yet well defined, it is known that the site at which metastasis develops can impact survival outcomes; therefore, the discovery of biomarkers indicative of the secondary seeding site(s) will be critical to evaluate the risk of site-specific recurrence [52–54]. Information from rapid autopsies will be fundamental to our understanding of these processes and in identifying site-specific biomarkers. Furthermore, the role of broader host and environmental factors in promoting metastasis, such as surgical removal of the primary tumor, age- and biomechanics-induced bone remodeling, as well as systemic stress hormones, are still emerging [55–60]. To robustly capture the long-term metastatic risk on this complex background, clinical studies will need to follow large cohorts over a sufficiently long period of time. This long-term data can then be used to understand the effects of these myriad factors on metastatic risk and will underpin the implementation of biomarkers in future precision medicine approaches.

## Understanding and modelling the dynamics of metastatic dissemination

### Research autopsies: an abundant resource to study human metastasis

Successfully dissecting and targeting the mechanisms that drive metastasis will depend on (1) a foundational understanding of metastasis mechanisms in patients, as well as (2) our ability to model metastasis in the laboratory. DTCs are difficult to detect, and secondary tumors at distant sites are often not surgically resected. Therefore, viable human tissue for researching metastasis mechanisms is scarce. Furthermore, as described above, assessing DTC/micrometastatic burden in patients and identifying features distinguishing indolent from aggressive DTCs has remained challenging. Research autopsies, also termed ‘warm’ or ‘rapid’ autopsies, of deceased cancer patients (1–6 h post-mortem) reveal the burden of disseminated disease at the end stages of disease and represent a valuable source of viable metastatic tissue [61]. The high integrity of tissues derived from research autopsies enables broad multi-omics analysis at the bulk and single cell resolutions to study cancer cells within the metastatic microenvironment. Importantly, research autopsies enable the establishment of patient-derived xenografts, cell lines and organoids for mechanistic studies [61]. Increased establishment of research autopsy protocols will require the multidisciplinary involvement of clinicians and researchers, together with the generosity of cancer patients and their families, in an effort that will continue to provide invaluable insight into metastatic burden and its drivers.

### Technological advances revealing tumor heterogeneity and spatial compartmentalization of the metastatic microenvironment

Emerging advances point to cellular heterogeneity and the microenvironment of primary tumors, pre-metastatic niches and metastatic sites as having a profound influence on the propensity and dynamics of metastatic dissemination. Interactions between cancer, stromal and immune cells as well as with the extracellular matrix spatiotemporally regulate metastasis [62, 63]. In this regard, the advent of single cell genomics and spatial profiling technologies are revolutionizing our understanding of cellular heterogeneity, cellular interactions and microenvironmental characteristics that support and promote metastasis.

Tumor heterogeneity and the evolution of highly metastatic subclones during the progression of heterogeneous tumors is thought to significantly contribute to metastatic propensity. Bulk analysis approaches have revealed specific mutational profiles and cellular states associated with polyclonal and monoclonal metastasis mechanisms in multiple cancer types [64–67]. More recently, single cell genomics approaches have been revolutionary in further revealing cellular subtypes within the broader heterogeneous tumor community that drive metastatic dissemination. For example, single cell RNA sequencing (scRNA-seq) of primary and metastatic patient-derived xenografts (PDXs) of mammary and lung tumors revealed an enrichment of stem-like/progenitor cell states in cancer cells within metastases as compared to primary tumors [68, 69]. Due to its single-cell resolution, single cell genomics will be particularly relevant in identifying mutationally- and transcriptionally-driven mechanisms of therapy resistance operating in subclonal cells that then outgrow treatment-sensitive subclones to drive continued tumor progression. For instance, single cell genomics was applied to chemo-refractory triple negative breast tumors, which revealed that resistant genotypes exist prior to neoadjuvant chemotherapy, and transcriptional programs that emerge in resistant cancer cell populations are induced by the treatment [70]. Using single-cell genomics in therapy-naïve primary lesions and paired, pan-resistant, anachronous secondary tumors in patient samples seems a challenging endeavor, but will significantly expand our understanding on therapy evasion mechanisms in metastasis.

Insights derived from early laser capture microdissection profiling of the tumor microenvironment have been accelerated by the application of multiplexed imaging (e.g. CODEX [71]), spatial transcriptomics (including the commercially available 10x Genomics Visium platform and emerging technologies slide-SEQ, non-destructive FISSEQ) and spatial proteomics analysis (e.g. imaging mass spectrometry) [72], together with the layering of these technologies on the more mature bulk and single cell genomics approaches. For example, multiplex imaging coupled with next generation sequencing has revealed that the close proximity of highly proliferative cancer cells with specific lymphocyte subtypes regulate immunological surveillance as metastases develop [73]. More detailed spatial characterization is now afforded by multiplexed ion beam imaging (MIBI)[74, 75], which harbors greater spectral depth than traditional fluorescence-based multispectral imaging, to enable the spatial identification of approximately 40 proteins within the microenvironment. This targeted technology has been integrated with spatial transcriptomics and single cell RNAseq in primary cutaneous squamous carcinoma to reveal the importance of spatially regulated cellular crosstalk nodes in tumorigenesis [76], demonstrating the potential of this approach in revealing key spatial relationships within tumors that could provide invaluable insight when specifically applied to metastatic disease.

Mass spectrometry imaging further extends the capacity of MIBI by mapping hundreds to thousands of metabolomic and proteomic analytes across tissue sections. Importantly, this technology identifies metabolic and proteomic features that cannot be obtained using the nucleotide-based spatial mapping technologies described above [77]. While metabolic and proteomic mass spectrometry imaging detection has been established for some time, recent improvements in sample preparation methods that more effectively preserve native tissue structure are unlocking the immense potential of this technology to reveal novel functional nodes of cell-cell and cell-matrix interactions governing metastatic processes [78]. This approach is particularly powerful in the burgeoning era of immunotherapy, where the interaction of cancer and immune cells is increasingly recognized to regulate metastasis. Recently, focused approaches have revealed a role for myeloid cells in activating dormant DTC proliferation through spatially compartmentalized laminin proteolysis [57, 58] and lipid metabolism [56]. Further spatial profiling of these environments using the wide lens conferred by mass spectrometry imaging may identify additional local nodes of cell-cell interactions that together regulate anti-tumor surveillance in a nuanced, microenvironment-specific manner. These advances in spatial technologies will be critical to provide fundamental answers to (1) how the immune milieu influences metastatic burden and (2) how these processes can be harnessed to control metastasis using existing and novel immunotherapy approaches. Insights into both metabolic and ECM remodeling within and surrounding tumor cells gained from mass spectrometry imaging also have the potential to reveal actionable stromal co-targeting approaches to improve treatment sensitivity [7, 79]. Overall, orthogonal spatial technologies have great potential to reveal novel mechanisms driving metastatic dissemination, which will underpin patient risk predictions and the development of metastasis-specific therapies.

### Modelling metastasis to predict risk

The identification of tumor features that support metastasis and predict metastatic risk in patients will facilitate improvements in vitro [80, 81] and in vivo models [82, 83] that recapitulate the dynamics of metastatic dissemination, colonization and outgrowth. Patient-derived xenografts and three-dimensional organoid cultures derived during treatment and from research autopsies more accurately reflect clinical treatment responses, and are currently being implemented as patient avatars in precision medicine programs to identify the most effective treatment for individual patients [84, 85]. As patient avatars, these models can be subjected to extensive drug screening in the laboratory to identify the most effective anti-tumor therapies that are likely to achieve a complete clinical response, thereby informing clinical practice to accelerate treatment. Improvements in animal models, including transplantable syngeneic mouse and human cancer lines, mouse xenografts, and genetically engineered- and humanized- mouse models are also being developed to mimic the behavior of human metastatic disease [86, 87]. While models of primary tumor behavior have received considerable attention and have significantly contributed to developments in cancer treatment, investment in developing metastasis-specific preclinical models should be prioritized for their capacity to reveal metastatic mechanisms that can be readily exploited therapeutically to improve patient survival. These technological developments are anticipated to most profoundly impact poor prognosis cancers and disadvantaged populations where stage IV diagnoses are more prevalent.

Data gathered from preclinical and clinical studies of metastasis dynamics are underpinning the implementation of mathematical modelling and artificial intelligence to build computational predictors of metastatic burden and therapy response [88]. These mathematical models also confer the opportunity to infer the actual stage of progression for a patient’s tumor at diagnosis, to predict their likely burden of disseminated disease and to assess their risk of developing overt metastases [89]. Importantly, mathematical models have validated the non-linear relationship between primary tumor size and survival, indicating that metastatic propensity is not simply a function of tumor size but that tumor intrinsic, extrinsic and host factors all contribute to metastatic progression [90]. Furthermore, clinically-informed mathematical models can define when metastases develop to enable earlier metastasis detection. For example, models of brain metastases from primary lung tumors have identified that DTCs in the brain remain dormant for approximately 5 months before their outgrowth, and that a further 12-19 months of secondary tumor growth occurs before metastasis is clinically diagnosed [91], thereby indicating that there is a significant window of opportunity to inhibit the outgrowth of disseminated cancer cells to the brain as well as improve the detection of early brain metastases. This temporal understanding, coupled with the predictive power of these models [89], provides the foundation to improve protocols for the early detection of metastatic disease and reveals opportunities to treat these patients earlier than current protocols allow. Mathematical models that are able to integrate the myriad host-, tissue- and niche-specific factors in metastatic dissemination and outgrowth will be key to understanding, as well as predicting, how these microenvironmental factors interact with intrinsic features of a patient’s primary tumor to drive the organotypic nature of metastasis [92]. Fueled by increased clinical data collection, these modelling approaches represent a step towards the clinical implementation of mathematical modelling as a predictive tool in precision oncology.

## Improving the treatment of metastatic disease

There are fundamental challenges in treating metastatic disease once it has been diagnosed. Tumor heterogeneity enables the persistence and expansion of treatment -refractory subclones at not only primary but also secondary sites. In addition, most current treatments are targeted to proliferative cell states, and therefore are less effective against quiescent metastatic disease prior to outgrowth. Disseminated disease, particularly when dormant, is commonly resistant to current standard-of-care therapies that target the primary tumor [93]. This enables residual disease to persist and re-emerge even after successful treatment of the primary tumor. Therefore, overcoming therapy resistance is paramount to prolonging the survival of metastatic patients.

Therapies that specifically target metastatic disease can be distinguished into two main categories: (1) treatments aimed at eradicating metastatic disease [29] and (2) treatments aimed at maintaining metastatic disease in a chronically dormant state [94].

### Treatments aimed at eradicating metastatic disease

Metastatic tumors have higher levels of resistance to systemic conventional chemotherapies compared with primary tumors and this presents a major hurdle to eradicating metastatic disease [95]. Resistance mechanisms to conventional chemotherapies can be due to chemotherapy-induced acquisition of de novo genomic alterations [96], phenotypic adaptation to cytotoxic stress [97] or result from the expansion of pre-existing clones [98]. These resistance traits can arise during both the latent and overt stages of metastases [93].

Given that conventional chemotherapies indiscriminately target rapidly dividing cells, it was assumed that DTCs in a quiescent or dormant state would be relatively insensitive to cytotoxic therapies. Therefore, one approach to sensitizing dormant cells to conventional chemotherapies is to drive them into a highly proliferative state. Approaches to stimulate the awakening of dormant cells, including G-CSF and acute IFN-alpha treatments [99, 100] or by driving epigenetic remodeling to transient drug-sensitive states (e.g. HDAC inhibition) [101], have improved the efficacy of cell cycle- and DNA damage response-dependent chemotherapeutic agents. However, these approaches carry a substantial risk of driving indiscriminate and uncontrollable metastatic outgrowth.

Recent data indicate that dormant cells can be targeted without the need to awaken them, thereby mitigating the significant risks associated with inducing them into a proliferative state. Rather than the quiescent state of dormant tumor cells giving rise to chemoresistance, these recent studies have identified cell-cell and cell-matrix interactions within the dormancy niche that protect DTCs from cytotoxic chemotherapies, independently of their proliferative state [9, 102]. Such studies are beginning to reveal potential strategies for targeting these interactions to re-sensitize metastatic tumors to current standard-of-care agents without inducing uncontrolled metastatic outgrowth. Some of these approaches have the potential for near-immediate translation into clinical protocols. For example, additional rounds of docetaxel chemotherapy following standard-of-care fluorouracil, epirubicin, and cyclophosphamide (FEC) treatment eliminated dormant tumor cells in the bone marrow of some breast cancer patients [103], highlighting how additional rounds of currently available chemotherapies may be used to effectively prevent metastasis. Conversely, emerging evidence points to the ability of standard-of-care chemotherapy to potentiate metastatic dissemination and drive the awakening of dormant DTCs in specific in vivo models [8]. This contrasts with evidence that neoadjuvant chemotherapy has been shown to be as efficacious as adjuvant chemotherapy in terms of relapse-free survival in some cancers, and therefore a more thorough understanding of the effects of therapy on metastatic dynamics in patients should inform treatment strategies in the future. Finally, identification of survival mechanisms exercised by DTCs has revealed potential therapeutic targets including autophagy [104], Srk and Mek1/2 in combination [105], and integrin-signaling [62, 102]. Overall, a more comprehensive understanding of these mechanisms is likely to reveal novel, specific targets of metastatic disease for further clinical development and provides hope that dormant DTCs can be eradicated without risking their uncontrolled proliferation.

Novel therapeutic strategies targeting metastasis are also emerging as our understanding of cancer cell-niche interactions develops. In bone metastases, identification of Jagged1 as a mediator of crosstalk between cancer cells and the bone microenvironment has led to the development of a new humanized anti-Jagged1 antibody that sensitizes bone metastases to chemotherapy [106]. Similarly, targeting integrin-mediated interactions in the perivascular niche of the bone microenvironment also prevents metastatic outgrowth in preclinical models [102]. Significant improvements in targeting metastatic disease will also rely on further development of nanoparticles and other chemotherapy carriers that can be targeted directly to specific secondary sites and pre-metastatic niches [17], thereby enabling combination therapies to simultaneously target multiple metastases. Defining how these niche-targeted treatments operate at different secondary sites and interact with each other will be critical in defining effective precision therapies.

### Treatments enforcing dormancy

Given the challenges in eliminating dormant DTCs, and the low penetrance by which dormant DTCs manifest as overt metastases, developing therapies that maintain DTCs in a dormant state is regarded as a promising therapeutic approach. Estrogen receptor (ER) antagonists such as tamoxifen, commonly used to treat ER+ breast cancer, are an example of anti-metastatic therapies administered in an adjuvant setting, although it remains unclear whether this treatment maintains dormancy or enables the elimination of dormant cells. Studies of tamoxifen treatment for 10 years found an additional reduction in metastatic recurrence rates when compared to the standard-of-care 5-year treatment regimen [107], highlighting the potential for this approach to achieve long term remission. Similarly, RANKL inhibitors, such as denosumab, have demonstrated utility in reducing bone resorption-driven activation of dormant DTCs in bone metastases of prostate cancer [108].

More recent proposed approaches to enforce metastatic dormancy include small molecules and monoclonal antibody therapies that drive DTCs into a dormant state. Induction of DTC dormancy has been achieved through CDK4/6 inhibition (e.g. Palbociclib) [109] or by inhibiting outside-in pro-proliferative signaling [95, 110]. Immunotherapy that harnesses immunological surveillance to control DTCs also has great potential to both eradicate and control metastatic disease since neutrophils and myeloid subtypes regulate microenvironmental cues that control dormancy [55–57]. For these reasons, immunotherapy is likely to become an essential component of metastasis management. Gaining a better understanding of the relationship between tissue-specific immune populations and DTCs, and how these interactions govern dormancy and outgrowth, will be essential to developing effective immunotherapies that control metastatic burden.

While treatments that eradicate or maintain cancer cell dormancy promise a future where metastatic latency can be effectively treated, therapies specifically targeting dormant metastatic disease are not yet clinically available. Fortunately, several clinical trials are ongoing with the explicit purpose of targeting dormancy and/or using DTCs or micrometastatic burden (for example, via detection of DTCs in bone marrow aspirates) as a readout of efficacy (Table 2). Nevertheless, substantial challenges remain in designing and completing clinical trials for metastasis-specific therapies. Regulatory frameworks around the world currently limit the feasibility of clinical trials with metastatic relapse at endpoints because the long time period required of such studies often exceeds that of intellectual property protection that underpins the financial support of the trial. Embarking on such a large and long trial is considered feasible when supported by substantial efficacy in the acute setting, and this hinders the development of cytostatic metastatic therapies that may not have substantial short term effects [97]. Furthermore, there must be a willingness from regulatory bodies to accept ctDNA and other biomarkers as clinical trial endpoints of metastasis, rather than the traditional RECIST-based progression-free survival readouts that encompass only overt metastases. The growing inclusion of disseminated disease burden as secondary endpoints in clinical trials will serve to (1) identify potential therapies for targeting dormancy after initial standard-of-care therapy has been completed and, (2) introduce the use of DTCs as a readout for therapeutic efficacy. Taken together, including disseminated disease burden in clinical trials is fundamental to developing more effective treatments against metastatic disease.

**Table 2:** Interventional clinical trials targeting metastatic disease

Approach	Cancer type	Secondary site	Therapy	Details	Trial ID
Detecting micrometastasis	Breast	Lymph nodes	Axillary lymph node dissection	Testing if omission of axillary lymph node dissection despite the presence of micrometastatic disease affects survival (SENOMIC, NEONOD2)	NCT02049632, NCT04019678
Preventing Metastasis	Lung	All	Pulmonary vein or arterial ligation prior to surgical resection of the tumor	Vein or arterial ligation during tumor resection to prevent metastatic spread; CTCs in the peripheral blood monitoring as primary endpoint	NCT03436329
Eliminate DTCs	Breast	Bone marrow	Trastuzumab (Her2-targeted mAb)	Treatment of Her2-positive DTCs in bone marrow in the setting of Her2-negative primary tumor	NCT01779050
Eliminate DTCs	Breast	Bone marrow	Docetaxel	Adjuvant docetaxel treatment following epirubicin frontline therapy to eliminate disseminated cancer cells in the bone marrow	NCT00248703
Eliminate DTCs	Breast (triple-negative)	Minimal residual disease	Sarilumab (IL6R mAb) + Capecitabine (anti-metabolite)	Treatment of triple negative breast cancer with minimal residual disease (EMPOWER)	NCT04333706
Eliminate dormant DTCs	Breast	Dormant cells in bone marrow	Avelumab (PD-L1 mAb) or hydroxychloroquine +/- palbociclib (CDK inhibitor)	Treatment to eliminate dormant breast cancer cells in the bone marrow identified in bone marrow aspirates (PALAVY)	NCT04841148
Prevent recurrence in Her2-positive breast cancer	Breast	All	Pertuzumab and transtuzumab (Her2-targeted therapies)	Treatment to inhibit recurrence (primary outcome) with adjuvant endocrine therapy and transtuzumab in stage I hormone receptor positive and Her2 positive breast cancer (ADEPT)	NCT04569747

## Concluding remarks

Metastatic disease remains the major cause of cancer death, yet in most cancer types we are only beginning to understand the processes that govern the dissemination of cancer cells from the primary tumor, their seeding to distant sites and their eventual outgrowth into overt metastases. It is a significant challenge to dissect the numerous host, environmental and microenvironmental factors that intersect with intrinsic features of cancer cells to spatiotemporally regulate metastatic progression. However, our ability to accurately detect DTCs and garner an accurate measure of metastatic burden in patients throughout treatment will underpin efforts to treat metastatic disease more effectively (Challenge 1). On this front, substantial strides have been made in improving the sensitivity of metastatic detection through machine-learning fueled pathological assessment and the use of liquid biopsy biomarkers to collectively detect CTCs in patients. Standardization of these approaches, together with increased access to research autopsies, will illuminate a hitherto opaque understanding of metastatic dynamics and will predict which patients are likely to develop metastatic recurrence (Challenge 2). This clinical data will continue to inform accurate in vitro and in vivo modelling of metastasis, to dissect the complex spatiotemporal interplay of cell intrinsic, microenvironmental and host factors (Challenge 3). Patient avatars developed from these models most accurately represent the patient’s treatment response and could become central to precision medicine frameworks that identify the most effective treatment for each individual. With further standardization, these tools may also prove fundamental to drug development pipelines for metastasis-targeted therapies. Dose-intensity modulation of existing therapies or novel therapies that eradicate or suppress metastases are being developed as biological mechanisms governing these processes are revealed (Challenge 4). Ultimately, translating these treatments to the clinic will require large, long-term clinical trials capable of supporting the complexity of individualized precision medicine protocols and with disseminated tumor burden, at both the micro- and macro-metastatic scales, as primary endpoints. Continued progress in overcoming these major challenges will require the collective interdisciplinary effort of researchers, clinicians, patients and funding agencies to transform metastatic cancer into a highly manageable and ultimately curable disease in all patients across all cancer types.

## Data Availability

Data sharing not applicable to this article as no datasets were generated or analyzed during the current study.
